# Recent Progress in PET and SPECT Myocardial Perfusion Imaging: From Radiopharmaceuticals to Imaging Innovations

**DOI:** 10.5812/ijpr-171684

**Published:** 2026-09-14

**Authors:** Elham Sattarzadeh Khameneh, Saeed Kakaei, Yashar Noshadi, Behrouz Alirezapour, Fariba Johari Daha

**Affiliations:** 1 Radiation Application Research School, Nuclear Science and Technology Research Institute (NSTRI) 11365 - 3486 Tehran Iran; 2 Medical Radiation, Nuclear Engineering, Shahid Beheshti University Tehran Iran

**Keywords:** PET Radiotracers, SPECT Radiotracers, Myocardial Perfusion Imaging, Cardiac Diagnostic Agents, Pyridazine-based Tracers, ^99^mTc-MIBI, Cardiac PET/SPECT Technology, Flurpiridaz, Myocardial Blood Flow Quantification

## Abstract

**Context:**

Cardiovascular diseases (CVDs) remain a leading cause of morbidity and mortality worldwide, underscoring the need for accurate, noninvasive diagnostic methods to support clinical decision-making and treatment planning. Myocardial perfusion imaging (MPI) with positron emission tomography (PET) and single-photon emission computed tomography (SPECT) plays a key role in the evaluation of myocardial ischemia and coronary artery disease. This review provides a comparative overview of PET and SPECT MPI, with particular emphasis on radiopharmaceuticals, technical advances, clinical applications, and the limitations of each modality.

**Evidence Acquisition:**

A systematic literature search was conducted in PubMed/MEDLINE, Scopus, Web of Science, and Google Scholar for studies published between 1990 and July 2026. Search terms included myocardial perfusion imaging, PET radiotracers, SPECT radiotracers, rubidium-82, nitrogen-13 ammonia, technetium-99m sestamibi, flurpiridaz, and relevant combinations of these terms using Boolean operators. Original research articles, clinical trials, and review articles addressing radiopharmaceutical development, image reconstruction techniques, quantitative imaging, and hybrid PET/CT and SPECT/CT technologies were included. Non-English publications, case reports, and studies lacking relevant original or comparative data were excluded. The reference lists of eligible articles were also manually reviewed. Radiotracers were compared with respect to pharmacokinetics, myocardial extraction fraction, target-to-background ratio, image quality, radiation dosimetry, and clinical utility.

**Results:**

SPECT MPI, particularly with ^99^mTc-sestamibi and ^99^mTc-tetrofosmin, remains widely used because of its established clinical infrastructure, relatively low equipment costs, broad availability, and ability to assess both myocardial perfusion and left ventricular function using gated imaging. Its principal limitations include lower spatial resolution, susceptibility to photon attenuation artifacts, and a limited capacity for absolute quantification of myocardial blood flow. PET MPI offers superior spatial and temporal resolution, routine attenuation correction, and quantitative measurement of myocardial blood flow and myocardial flow reserve. Established PET tracers, including ^82^Rb and ^13^N-ammonia, generally provide higher diagnostic accuracy than conventional SPECT; however, their short physical half-lives require generator-based or on-site cyclotron production and may limit the feasibility of exercise stress protocols. Emerging ^18^F-labeled tracers, particularly flurpiridaz, offer several advantages, including a longer half-life suitable for centralized production and distribution, improved image contrast, exercise stress imaging, rapid extracardiac clearance, and a more linear relationship between myocardial tracer uptake and blood flow over a broad flow range. Recent phase III studies have further supported the diagnostic performance of flurpiridaz.

**Conclusions:**

PET and SPECT play important and complementary roles in noninvasive myocardial perfusion imaging. SPECT remains the most widely available and cost-effective technique, whereas PET provides superior image quality, attenuation correction, and quantitative assessment of myocardial blood flow. Ongoing advances in radiopharmaceutical development, hybrid imaging systems, reconstruction methods, and quantitative software are expected to further enhance the diagnostic and prognostic value of MPI and support increasingly personalized approaches to cardiovascular care.

## 1. Context

The heart is a vital muscular organ that serves as the central pump of the circulatory system. It is enclosed within a fibrous sac called the pericardium (Figure 1). The heart has four chambers, including two atria and two ventricles, separated by septal walls. The right atrium receives deoxygenated blood from the body and pumps it to the right ventricle, which then pumps blood to the lungs. The left atrium receives oxygenated blood from the lungs and pumps it to the left ventricle, which distributes blood throughout the body (1-3).

**Figure 1.**
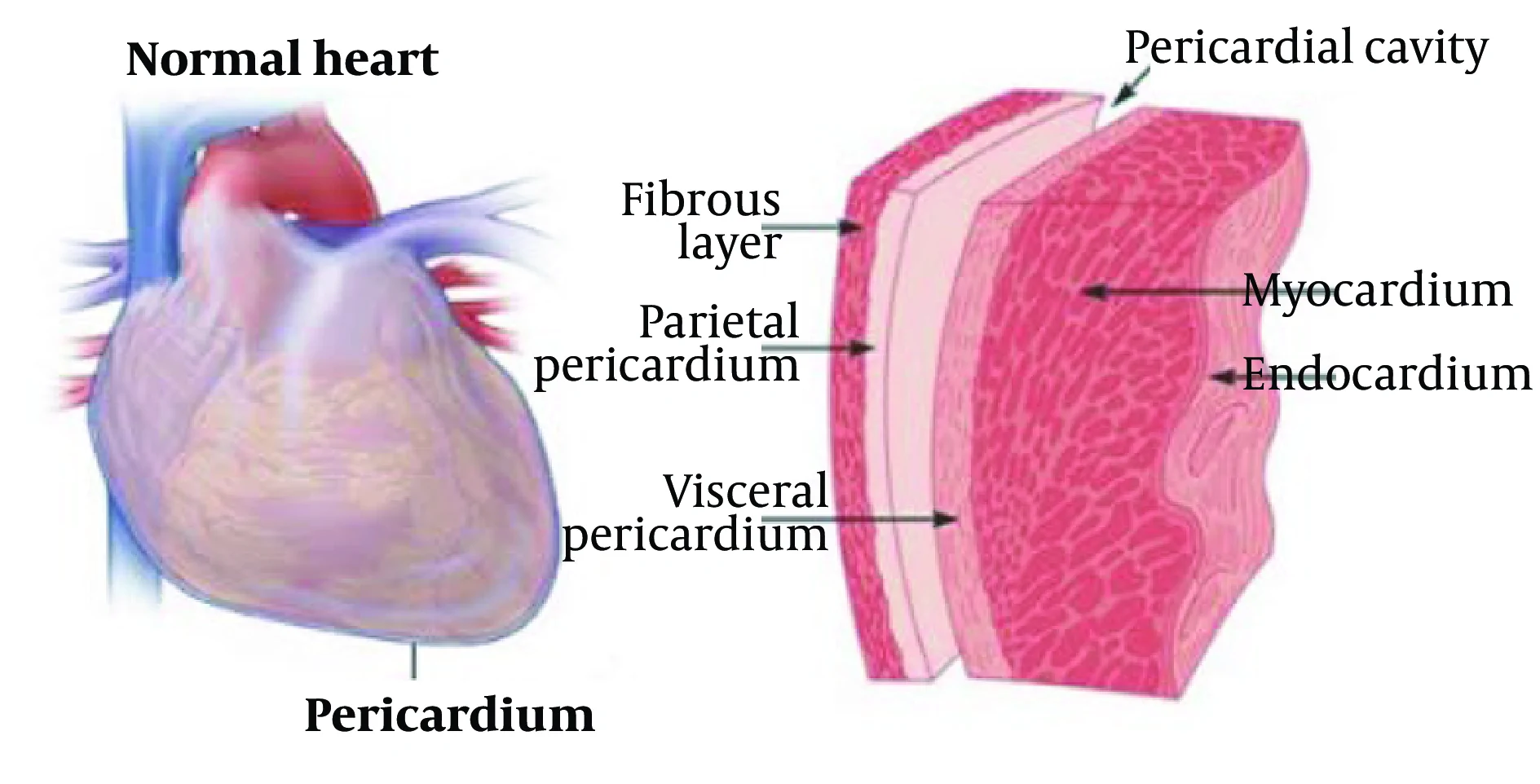
Pericardial sac and different parts of the pericardium

The coronary arteries supply blood to the heart muscle, and blockages in these arteries are a leading cause of myocardial infarction and heart failure. Cardiovascular diseases (CVDs) are disorders that affect the heart and blood vessels. Coronary artery disease (CAD) is the most common type and occurs when the coronary arteries become narrowed or blocked by atherosclerotic plaque buildup, potentially leading to myocardial infarction, heart failure, or arrhythmias (4, 5). Additional CVDs include angina pectoris (chest pain due to reduced myocardial blood flow), heart failure (impaired pumping function), hypertension, cardiomyopathy, cardiac arrhythmias, valvular heart disease, and peripheral artery disease. Despite medical advances, CAD remains the leading cause of death worldwide (6), and accurate diagnostic techniques are essential for disease identification and management (7). Myocardial perfusion imaging (MPI) has evolved substantially over the past four decades (8, 9) as a noninvasive method for assessing coronary blood flow and identifying perfusion abnormalities (10). Currently, two major molecular imaging techniques are widely used for cardiovascular assessment: single-photon emission computed tomography (SPECT) and positron emission tomography (PET) (8, 11). These modalities provide noninvasive assessment of perfusion and myocardial function (12) and support disease staging and treatment planning (13).

SPECT is the standard and most widely used nuclear imaging method and has been broadly available in most hospitals since the early 1990s (14). Nuclear cardiology has been a clinical specialty for approximately four decades, with the first use of radioisotopes to study circulatory processes reported in 1927 (15). Labeled molecules enable visualization, measurement, and tracking of metabolic processes, thereby distinguishing between healthy and diseased tissue (15). Recent advances in physics and related technologies have improved all aspects of medical imaging, particularly nuclear cardiology. Nuclear medicine imaging is now essential for diagnosing and managing cardiac disease, assessing blood supply during exercise and rest, identifying regions of poor perfusion, and detecting viable tissue in patients with prior myocardial infarction (16). The development of 2D and 3D imaging devices has created new opportunities for medical imaging research (16).

### 1.1. Scope and Objective

This review provides a comprehensive comparative overview of PET and SPECT myocardial perfusion imaging, with a detailed focus on radiopharmaceuticals, technical innovations, clinical applications, and limitations. The review addresses the following questions: 1) what are the key characteristics of current SPECT and PET perfusion tracers, and how do they compare? 2) what are the advantages and limitations of each modality in clinical practice? 3) what emerging tracers and technologies may overcome current limitations? The comparative analysis emphasizes radiopharmaceutical performance, clinical utility, and practical considerations for implementation in cardiovascular imaging.

## 2. Evidence Acquisition

A systematic literature search was performed in PubMed/MEDLINE, Scopus, Web of Science, and Google Scholar to identify studies published between 1990 and July 2026. Search terms included myocardial perfusion imaging, PET radiotracers, SPECT radiotracers, rubidium-82, nitrogen-13 ammonia, technetium-99m sestamibi, flurpiridaz, and relevant Boolean combinations. Original research articles, clinical trials, and review articles addressing radiopharmaceutical development, image reconstruction techniques, quantitative imaging, and hybrid PET/CT and SPECT/CT technologies were included. Non-English publications, case reports, and studies lacking relevant original or comparative data were excluded. Reference lists of eligible articles were also reviewed manually. Radiotracers were compared with respect to pharmacokinetics, myocardial extraction fraction, target-to-background ratio, image quality, radiation dosimetry, and clinical utility.

## 3. Results

### 3.1. Myocardial Perfusion SPECT Radiopharmaceuticals

Selection of an appropriate radiopharmaceutical is critical in nuclear cardiology, as the radionuclide’s physical half-life should align with the duration of the physiological process under investigation (7, 17).

#### 3.1.1. Thallium-201 Chloride (^201^Tl)

Thallium-201 (^201^Tl, half-life 73 hours) was the first clinically adopted SPECT MPI tracer, introduced in the 1970s. It is a potassium analog transported across cell membranes via the Na^+^/K^+^ ATPase pump, with rapid myocardial extraction and subsequent redistribution reflecting myocardial viability (18-20). ^201^Tl is cyclotron-produced and has a long physical half-life, requiring lower administered activities to minimize radiation exposure. However, its low-energy photons (71 - 80 keV) result in longer imaging times and limited image quality due to photon attenuation and scatter, particularly in obese patients (21, 22). ^201^Tl distribution is proportional to organ blood flow. Following intravenous injection, normal myocardium rapidly extracts the tracer (5 - 8% remains in blood at 5 minutes), with slow excretion via feces and urine (21, 22).

As a monovalent cation, ^201^Tl behaves as a potassium analog and is actively transported into viable myocytes. Injection occurs at peak exercise or during pharmacological stress. Initial perfusion defects may indicate ischemia or non-viable myocardium. After cessation of stress, ^201^Tl redistributes, and ischemic areas may demonstrate reversible defects (filling in on delayed images) (23).

#### 3.1.2. Thallium-201 Disadvantages

^201^Tl provides good contrast between normal and ischemic regions. However, its 73-hour physical half-life and approximately 10-day biological half-life result in a long effective half-life of about 56 hours. Significant accumulation occurs in the liver, kidneys, and skeleton, contributing to an effective dose of 34.5 mSv per 150 MBq (Table 1). The relatively high absorbed dose limits administered radioactivity to less than 150 MBq, potentially compromising image quality (23). ^201^Tl decays by electron capture to ^201^Hg, emitting low-energy X-rays (75 - 80 kV) that are captured during imaging (10).

**Table 1.** ^201^Tl-Cl Tracer Features for Myocardial Perfusion Blood Flow

Tracer	^201^Tl
**Chemical structure**	^201^Tl^+^
**Type of tracer**	Metal cation
**Production**	Cyclotron
**Half-life**	73 h
**Scan Duration (rest and stress)**	4 h
**Intravenously administered activity (MBq)**	74 - 148
**Effective Dose (mSv/MBq)**	0.23
**Approval year (US FDA / EU)**	1977 / 1980

#### 3.1.3. Technetium-99m-Based Tracers

^99^mTc-tetrofosmin is a cationic technetium-99m complex in the +5 oxidation state, formulated as [^99^mTc(tetrofosmin)_2_O_2_]^+^ (24, 25). It is commercially available as a lyophilized kit (Myoview, GE Healthcare) containing 0.23 mg tetrofosmin and excipients. Labeling is performed by adding sodium pertechnetate (^99^mTc) from a ^99^Mo/^99^mTc generator to the vial and incubating for 15 minutes at room temperature. Radiochemical purity must exceed 90% (24, 25).

Cardiac uptake of ^99^mTc-tetrofosmin occurs via passive diffusion without redistribution. Following intravenous injection, myocardial uptake reaches a maximum within 5 minutes. Approximately 66% of the injected dose is excreted within 48 hours (40% urine, 26% feces). ^99^mTc-tetrofosmin is used for myocardial perfusion imaging under stress and rest conditions to identify reversible ischemia and infarcted tissue (Figure 2). It effectively detects perfusion changes due to pharmacological stress (adenosine) in CAD patients. ^99^mTc-tetrofosmin rapidly clears from blood (5% remains at 10 minutes), with myocardial uptake of approximately 1.2% at peak, minimal redistribution (~1% at 2 hours), and rapid hepatic and pulmonary clearance (26-28). It is also used to evaluate left ventricular function.

**Figure 2.**
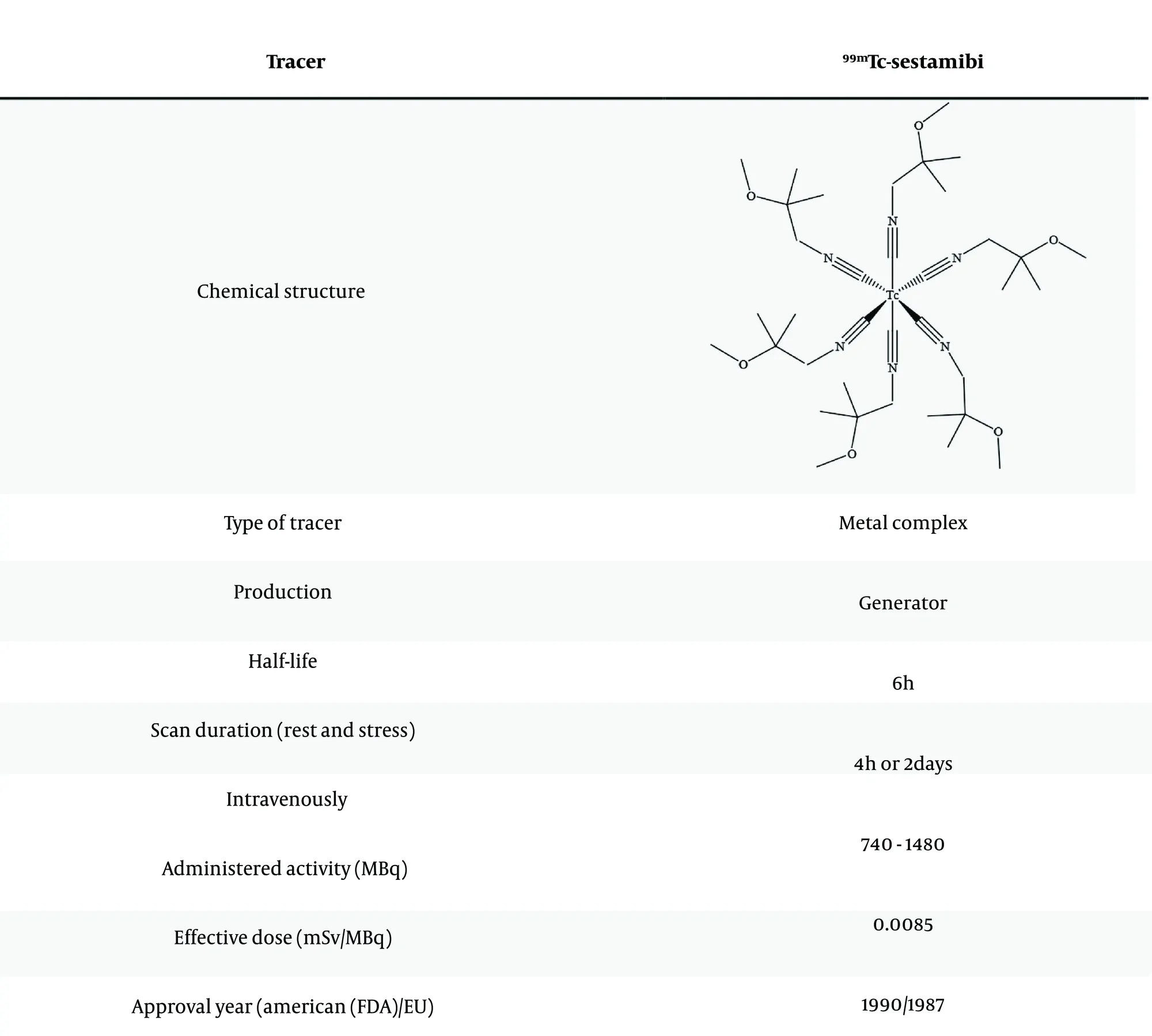
^99^mTc-tetrofosmin tracer features for myocardial perfusion blood flow

#### 3.1.4. ^99^mTc-Sestamibi (MIBI)

^99^mTc-Methoxyisobutylisonitrile (^99^mTc-MIBI, sestamibi) is a cationic, lipophilic complex with a half-life of 6.01 hours. It consists of a ^99^mTc atom in the 1^+^ oxidation state coordinated to six MIBI ligands. The isonitrile groups form a complex with reduced ^99^mTc. Due to the volatility and instability of isonitrile, MIBI is stabilized as [Cu(MIBI)_4_]BF_4_, which decomposes during high-temperature radiolabeling. Commercial kits (Cardiolite, CardioTop, Technescan Sestamibi) contain [tetrakis (2-methoxy-2-methylpropyl-1-isocyanide) copper (1^+^)] tetrafluoroborate with stannous chloride (reducing agent), L-cysteine hydrochloride, sodium citrate (buffer), and mannitol. Labeling involves adding sodium pertechnetate (^99^mTc) and heating in a boiling water bath for 10 minutes (7).

^99^mTc-sestamibi is primarily used to diagnose myocardial perfusion abnormalities, particularly ischemia and infarction, and to evaluate myocardial function using first-pass radionuclide ventriculography. It enters myocardial cells via passive diffusion, localizes to mitochondria, and accumulates proportionally to myocardial perfusion with slow washout (7). Under stress, > 3% of the injected dose accumulates in the myocardium. ^99^mTc-sestamibi acts as a calcium analog, is trapped in mitochondria, and does not redistribute, unlike ^201^Tl. It provides good perfusion imaging with rapid blood clearance, high myocardial extraction, and limited uptake in the thyroid and salivary glands (Figure 3).

**Figure 3.**
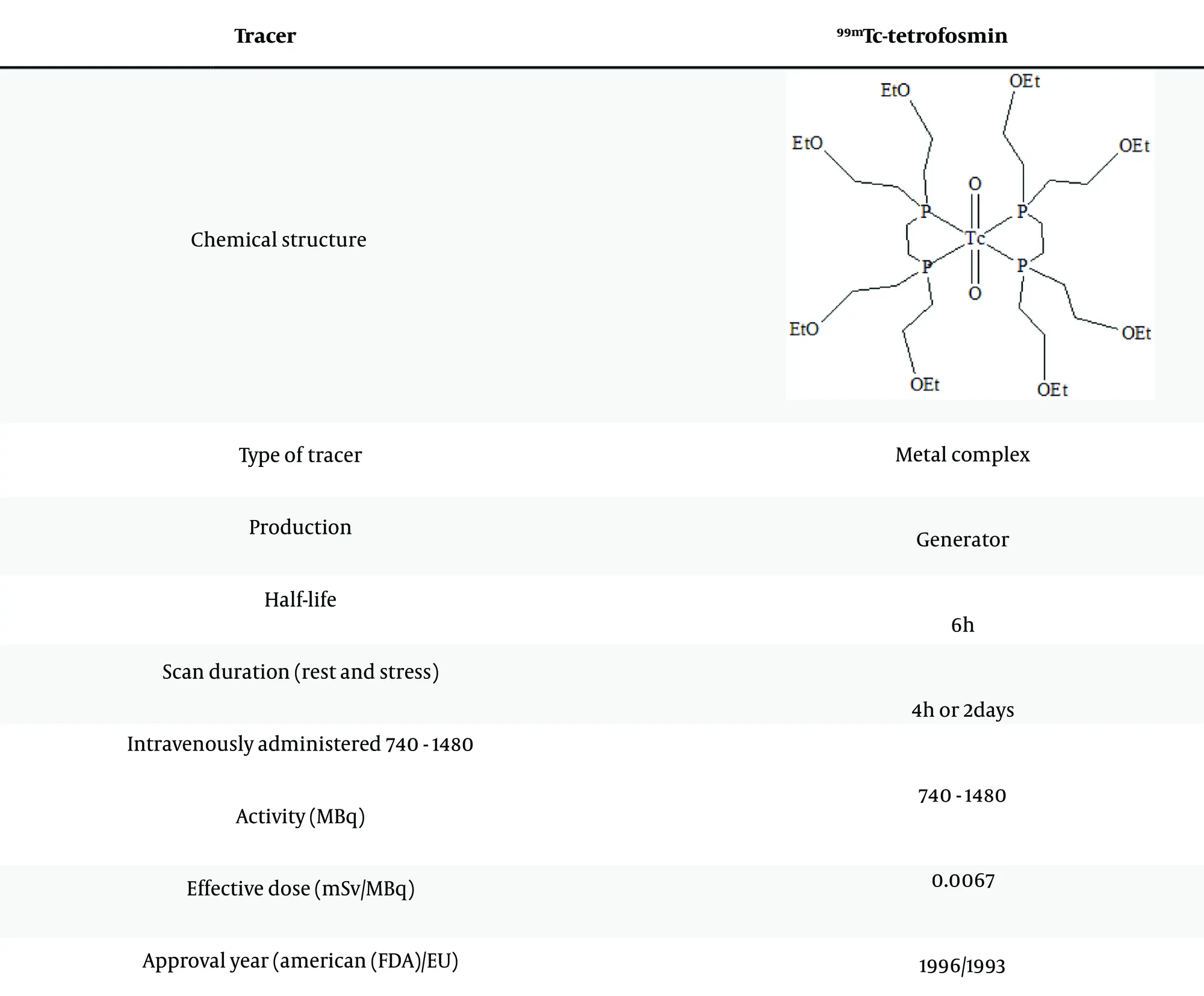
^99^mTc-sestamibi tracer features for myocardial perfusion blood flow

#### 3.1.5. ^123^I-Meta-Iodobenzylguanidine (mIBG)

Heart failure (HF) is characterized by sympathetic nervous system activation (29-31, 32). Metaiodobenzylguanadine (mIBG), or iobenguane, originally developed as a pheochromocytoma imaging agent, accumulates in address an effective dose of 2.4 mSv in adults, with doses up to 55 - 111 MBq/kg possible with new gamma cameras. MIBG is excreted primarily via the kidneys. Distribution in SPECT studies is similar to perfusion tracers (e.g., ^123^Tc-tetrofosmin).

#### 3.1.6. ^99^mTc-HYNIC-Annexin V

Apoptosis (programmed cell death) plays a significant role in cardiovascular disease pathogenesis (33, 34). During early apoptosis, phosphatidylserine appears on the outer cell membrane. Annexin V, a 319-amino acid human protein, binds with high affinity to phosphatidylserine (35, 36). ^99^mTc-Annexin V is the most widely used radiopharmaceutical for apoptosis imaging (37).

Apoptosis imaging with ^99^mTc-Annexin V, combined with perfusion imaging (^99^mTc-sestamibi, ^99^mTc-tetrofosmin, or ^201^Tl), has been used in patients with myocardial infarction after percutaneous coronary angioplasty (PTCA) (38, 39). Increased ^99^mTc-Annexin V uptake at infarct sites correlates with myocardial hypoperfusion, indicating apoptosis. The absence of ^99^mTc-Annexin V uptake at 4 days post-infarction, corresponding to reduced ^99^mTc-sestamibi defect size, suggests reversible myocardial damage rather than necrosis (Table 2) (39, 40).

**Table 2.** Comparison of the Main Blood-Flow SPECT Tracers

Tracer	Physical Half-life	Myocardial Extraction (%)	Clearance Half-Life
**Thallium-201**	73 h	85	slow
**^99^mTc-sestamibi**	6 h	65	Very slow
**^99^mTc-tetrofosmin**	6 h	60	Very slow

#### 3.1.7. Summary of SPECT Tracer Clinical Status

Currently, ^99^mTc-sestamibi and ^99^mTc-tetrofosmin are the most widely used SPECT MPI tracers, with ^201^Tl used less frequently due to its radiation dose and imaging limitations. ^123^I-mIBG and ^99^mTc-Annexin V are used for specialized applications (sympathetic innervation and apoptosis imaging, respectively) rather than routine perfusion assessment.

#### 3.1.8. Extracardiac Activity and Artifact Reduction in SPECT

A significant limitation of SPECT MPI is interference from extracardiac activity, particularly from the liver, bowel, and stomach (41, 42). Hepatobiliary excretion of ^99^mTc-based agents often results in liver and bowel activity, while gastric activity may arise from duodenal reflux or uptake of free ^99^mTc-pertechnetate by the gastric mucosa. These artifacts can mimic or obscure inferior wall perfusion defects, reducing diagnostic accuracy. Strategies to minimize extracardiac activity include: 1) patient hydration and fatty meal consumption to promote hepatobiliary clearance; 2) prone imaging to shift extracardiac activity away from the inferior wall; 3) carbonated beverage ingestion to displace gastric activity; and 4) delayed imaging when necessary (43, 44).

#### 3.1.9. CZT Detector Technology in Modern SPECT

Cadmium-zinc-telluride (CZT) semiconductor detectors represent a significant technological advance in SPECT imaging. Unlike conventional NaI (Tl) scintillation detectors with photomultiplier tubes, CZT detectors directly convert photons to electrical signals, providing: 1) superior energy resolution (approximately 5 - 6% at 140 keV vs. 9 - 10% for NaI (Tl)); 2) markedly improved sensitivity (up to 10-fold higher); 3) reduced acquisition times (down to 5 - 10 minutes); and 4) potential for significant dose reduction (up to 50%). CZT-based SPECT systems have demonstrated improved diagnostic accuracy for CAD detection, particularly in challenging patient populations such as obese individuals and women with breast attenuation. The technology enables dynamic SPECT imaging and has the potential to facilitate absolute myocardial blood flow (MBF) quantification, although this remains an area of active investigation.

### 3.2. Myocardial Perfusion PET Tracers

Positron emission tomography (PET) offers significant advantages for MPI, including superior spatial and temporal resolution, routine attenuation correction, and the capacity for absolute quantification of MBF. The field is evolving through the integration of hybrid PET systems, advanced computational techniques, and the development of novel, mechanism-specific radiopharmaceuticals aimed at overcoming longstanding limitations in cardiac imaging (45).

#### 3.2.1. Positron Range and Its Impact on Spatial Resolution

The effective spatial resolution of PET imaging is limited by the positron range, the distance a positron travels before annihilation. This physical phenomenon causes misregistration of annihilation events relative to the original tracer location. Positron range varies substantially among radionuclides based on their maximum positron energy: ^18^F (0.64 MeV, mean range 1.03 mm) provides the highest spatial resolution; ^13^N (1.19 MeV, mean range 2.53 mm) offers intermediate resolution; ^15^O (1.73 MeV, mean range 4.14 mm) has moderate resolution; and ^82^Rb (3.38 MeV, mean range 8.6 mm) has the lowest resolution. This range-dependent degradation means that ^82^Rb PET images have inherent blurring that can limit detection of small perfusion defects, whereas ^18^F-labeled tracers achieve near-optimal spatial resolution. Modern PET/CT systems with time-of-flight capability partially compensate for these effects, but the fundamental limitations remain.

Accurate attenuation correction (AC) is essential for quantitative PET and SPECT imaging. PET offers routine, robust AC using transmission scans or CT-based attenuation maps. In PET/CT, the CT image provides a high-quality attenuation map that is co-registered with the PET emission data, enabling accurate correction for photon attenuation in heterogeneous tissues. This is particularly important in obese patients and those with breast or diaphragm attenuation. In contrast, SPECT AC is more challenging; conventional methods use external radioisotope sources (e.g., ^153^Gd line sources) or CT-based maps on SPECT/CT systems. However, SPECT AC is limited by the non-overlapping energies of the emission and transmission photons, potential misregistration, and the need for correction of scatter and tissue heterogeneity. The inability to routinely perform accurate AC has historically limited SPECT’s diagnostic specificity compared with PET.

#### 3.2.2. Absolute Quantification of Myocardial Blood Flow With PET

A unique advantage of PET MPI is the ability to quantify absolute MBF (mL/g/min) using dynamic imaging and tracer kinetic modeling. This contrasts with SPECT, which provides only semi-quantitative indices such as the summed stress score (SSS), summed rest score (SRS), and summed difference score (SDS). PET MBF quantification uses compartmental models that account for tracer delivery, extraction, and retention. For ^13^N-ammonia, a two-tissue compartment model is typically employed:

dCm(t)/dt = K_1_ · C_a_(t) – k_2_ · C_m_(t) – k_3_ · C_m_(t) + k_4_ · C_t_(t)

dCt(t)/dt = k_3_ · C_m_(t) – k_4_ · C_t_(t)

where C_a_ is the arterial input function, C_m_ is free ammonia in myocardium, C_t_ is metabolically trapped ammonia, and K_1_, k_2_, k_3_, k_4_ are rate constants. For ^82^Rb, a simpler one-compartment model is often sufficient:

dC_m_(t)/dt = K_1_ · C_a_(t) – k_2_ · C_m_(t)

Myocardial flow reserve (MFR = MBF_stress_/ MBF_rest_) provides a measure of the hemodynamic significance of coronary stenosis. Quantitative MBF has demonstrated superior diagnostic performance compared with relative perfusion assessment, particularly for detecting balanced ischemia and multivessel disease. However, accurate quantification requires careful attention to: 1) arterial input function extraction; 2) correction for partial volume effects; 3) spillover between the blood pool and myocardium; and 4) appropriate model selection and parameter estimation.

#### 3.2.3. Partial Volume Effects and Spillover in PET MPI

Partial volume effects (PVE) arise from the limited spatial resolution of PET, causing underestimation of activity in small structures and spillover between adjacent regions. In cardiac PET, this is particularly important for the left ventricular cavity (blood pool) and myocardial wall. PVE and spillover affect tracer kinetic modeling and MBF quantification. Different tracers have different PVE characteristics: ^15^O-water (long positron range) shows significant spillover between blood and myocardium, requiring complex correction models; ^82^Rb (intermediate range) has moderate PVE; and ^18^F-labeled tracers (short range) minimize PVE. Correction methods include recovery coefficient approaches, geometric transfer matrix methods, and partial volume correction algorithms integrated into kinetic models. Failure to account for PVE and spillover can lead to systematic errors in MBF estimates, particularly in thin-walled myocardial segments.

#### 3.2.4. Dynamic Framing Challenges for Short-Lived Tracers

Generating the arterial input function for MBF quantification requires dynamic PET acquisition with short frame durations. For ^82^Rb (76-second half-life), this is challenging because: 1) early frames must be very short (1 - 3 seconds) to capture the rapid peak; 2) count statistics are limited, increasing noise; 3) rapid decay requires precise timing; and 4) the input function is highly dependent on accurate bolus characterization. Typical framing protocols for ^82^Rb include 30 frames: 12 × 1 - 3 seconds, 6 × 5 seconds, 4 × 10 seconds, 3 × 20 seconds, and 5 × 60 seconds. For ^13^N-ammonia (10-minute half-life), framing is less demanding, with protocols including 6 × 5 seconds, 4 × 15 seconds, 4 × 30 seconds, and 6 × 60 seconds. The quality of the arterial input function directly affects MBF quantification accuracy; poor count statistics or improper framing can introduce significant errors, particularly at high flow rates during pharmacological stress.

#### 3.2.5. Rubidium-82 (^82^Rb) and Nitrogen-13-Ammonia (^13^N-Ammonia)

Rubidium-82 (^82^Rb) and nitrogen-13-ammonia (^13^N-ammonia) are the only PET radiotracers currently FDA-approved for MPI, with ^15^O-water limited to research applications (7, 46). These tracers have short half-lives: 76 seconds (^82^Rb), 2 minutes (^15^O-water), and 10 minutes (^13^N-ammonia), requiring either on-site cyclotrons or generators. ^82^Rb, a potassium analog, is actively transported into the myocardium via the Na^+^/K^+^ ATPase pump. Its short half-life and generator availability enable rapid imaging protocols with favorable dosimetry (47). Despite a relatively low myocardial extraction fraction (approximately 65%), ^82^Rb has been validated for MBF measurement using single-tissue compartment modeling (48).

^13^N-ammonia diffuses across cell membranes as NH_3_, equilibrates with NH_4_^+^, and is trapped in cells via conversion to ^13^N-glutamine, glutamic acid, and carbamoyl phosphate (49). It has a high first-pass extraction fraction of approximately 80% and minimal redistribution, offering advantages over ^82^Rb for MBF quantification. Its small positron range (2.53 mm) provides good spatial resolution and defect detection (7, 46). However, the need for an on-site cyclotron and short half-life (10 minutes) make exercise stress impractical in clinical settings. Different techniques for absolute quantification of dynamic ^13^N-ammonia PET data have been validated, with single-tissue or two-tissue compartment models used (48, 50, 51). Model selection affects MBF estimates, highlighting the importance of standardized protocols.

#### 3.2.6. Oxygen-15-Water (^15^O-Water)

^15^O-water is considered the reference standard for quantitative MBF measurement due to its freely diffusible nature and linear relationship between first-pass extraction and perfusion (7, 46, 49). Its short half-life (2.04 minutes) requires on-site cyclotron synthesis, limiting clinical use. Additionally, the long positron range (4.14 mm) and poor myocardial-to-background ratio make visual image interpretation challenging. Despite recent data supporting its clinical use (52), ^15^O-water is not FDA-approved for routine clinical use, being primarily restricted to research applications.

^15^O-water PET has been used as a reference standard for MBF quantification, against which other methods have been validated (53-58). The rest and stress protocol can be completed in approximately 30 minutes using modern PET/CT scanners (59). A parametric perfusable tissue index, derived from a single ^15^O-water PET/CT scan, has been developed as a marker of myocardial viability (60), potentially increasing clinical utility. However, the short half-life precludes exercise stress imaging, and the low myocardial accumulation limits visual assessment capabilities. The US FDA and Japanese Ministry of Health, Labour, and Welfare have not approved ^15^O-water for clinical use (61).

#### 3.2.7. Rubidium-82 (^82^Rb): Detailed Discussion

^82^Rb, generated by the ^82^Sr/^82^Rb generator (CardioGen-82), has practical advantages, including independence from an on-site cyclotron and a generator lifespan of 4 - 5 weeks (62, 63). The ultra-short half-life (76 seconds) enables rapid sequential studies, and radiation exposure is lower than ^99^mTc-based SPECT (effective dose 2 - 3.7 mSv vs. 5 - 10 mSv) (64, 65). ^82^Rb can show subdiaphragmatic activity in the stomach and pancreas due to Na^+^/K^+^-ATPase-mediated uptake, which can interfere with inferior wall interpretation (42-44, 66). Strategies to reduce this artifact include water ingestion, prone imaging, and fatty meals. The availability of daily generator elutions and rapid acquisition times (10-minute intervals) allow imaging of up to 10 - 15 patients per day (67). The ^82^Sr/^82^Rb generator can be operated for 28 - 42 days, with potential extension to 60 days to reduce per-patient cost.

^82^Rb PET offers superior count density and uniformity compared with SPECT, with higher interpretive certainty and reader agreement (68). Because PET cameras do not require collimators, they provide better sensitivity and resolution. The primary advantage of ^82^Rb PET is accurate MBF and flow reserve quantification, which remains a challenge for SPECT (Table 3).

**Table 3.** Characteristics of Various Cardiac PET Perfusion Tracers

Characteristic	^18^F-Flurpiridaz	^13^N-Ammonia	^15^O-Water	^82^Rb
**Half-life (min)**	109	9.96	2.06	1.25
**Production**	Regional cyclotron	Onsite or nearby cyclotron	Onsite cyclotron	Generator
**Positron range (mm)**	1.03	2.53	4.14	8.60
**Image resolution**	Highest	Intermediate-high	Intermediate	Lowest
**Myocardial extraction fraction (%)**	94	80	100	65
**Perfusion defect contrast**	Highest	Intermediate	Intermediate ^a^	Lowest
**Pharmacologic stress imaging protocol**	Feasible	Feasible	Feasible	Feasible
**Treadmill exercise imaging protocol**	Feasible	Feasible but not practical	Not feasible	Not feasible

^a^ Theoretically, a 100 percent myocardial extraction fraction of ^15^O-water should produce the most incredible perfusion defect contrast. On the other hand, a weak myocardial-to-background ratio reduces the contrast defect.

### 3.3. Stress Protocols for Myocardial Perfusion Imaging

Stress MPI can be performed using either exercise (treadmill or bicycle) or pharmacological agents. The choice of stress protocol significantly affects hemodynamic parameters and MBF quantification:

Exercise produces physiological hyperemia through increased myocardial oxygen demand, with peak MBF typically reaching 3 - 5 mL/g/min. It provides additional functional information (symptoms, ECG changes, exercise capacity) and patient satisfaction. Limitations include the inability to achieve target heart rate in deconditioned patients and the lack of a steady-state MBF during imaging. Exercise stress is only feasible with tracers that allow post-exercise injection (^201^Tl, ^99^mTc-agents, and potentially ^18^F-labeled agents).

A non-selective vasodilator that produces potent coronary hyperemia with minimal hemodynamic changes, achieving MBF of 4 - 6 mL/g/min. Side effects include chest pain, dyspnea, flushing, and atrioventricular block. Adenosine requires continuous infusion and is contraindicated in reactive airway disease and advanced heart block. Regadenoson: A selective A_2A_ adenosine receptor agonist with rapid bolus administration and fewer side effects, providing comparable hyperemia to adenosine. Regadenoson has largely replaced adenosine in clinical practice because of ease of use and improved patient tolerability.

A β-1 agonist that increases heart rate and contractility, producing flow reserve comparable to exercise. It is used in patients unable to exercise and with contraindications to vasodilators. Dobutamine increases blood pressure, which may affect MBF quantification.

Stress protocol selection influences MBF quantification: vasodilators produce more uniform hyperemia and are better suited for MBF reserve assessment, whereas dobutamine stress provides functional information similar to exercise. For PET MBF quantification, vasodilators are typically preferred because of the steady-state MBF required for accurate measurement. Current guidelines recommend pharmacologic stress for PET MPI, although ^18^F-labeled tracers may enable exercise protocols.

Despite these advantages, PET is not universally used for clinical myocardial perfusion imaging for several reasons. Financial factors include higher equipment costs for PET/CT scanners, continuous generator expenses for ^82^Rb, and the need for cyclotron facilities for ^13^N-ammonia. Clinical limitations include short tracer half-lives that preclude exercise stress, gastrointestinal background activity, and variable myocardial extraction characteristics. However, emerging ^18^F-labeled tracers aim to address these limitations.

### 3.4. The Emergence of Fluorine-18-Labeled Tracers

Over the past several decades, substantial effort has been devoted to developing ^18^F-labeled PET MPI tracers. ^18^F (half-life 110 minutes, positron range 1 mm) offers advantages that may overcome current limitations (69-71):

Long half-life enabling centralized production and distribution, comparable to ^18^F-FDG logistics (72, 73).

Lowest positron range among MPI PET tracers, providing the highest spatial resolution (72, 74).

Lower positron energy and higher positron fraction allowing reduced administered activities (75).

Half-life suitable for delayed imaging protocols (76).

Potential for exercise stress protocols, unlike short-lived PET agents (69).

^18^F-labeled phosphonium cations are classified by mitochondrial targeting mechanisms: 1) voltage-dependent cations that penetrate mitochondria based on membrane potential; and 2) mitochondrial complex-1 (MC1) inhibitors (7, 77).

Phosphonium analogues, including ^18^F-FBnTP, ^18^F-FTPP, and ^18^F-FPTP, have shown rapid myocardial uptake and retention (78-80).

^18^F-FBnTP, a lipophilic cation, accumulates in cardiac mitochondria (81). In canine studies, left ventricular activity plateaued at 5 minutes and remained detectable at 90 minutes, with minimal metabolite formation and high heart-to-lung ratios (12:1 at 5 minutes) compared with SPECT agents. ^18^F-FBnTP demonstrated superior diagnostic performance to ^99^mTc-tetrofosmin in detecting coronary stenoses in canine models (82-84). In Wistar rat studies, ^18^F-FBnTP showed no washout or redistribution after transient coronary occlusion, with defects matching histology (85). ^18^F-FBnTP has been used as a reference perfusion marker in myocarditis studies (86), although human data remain limited.

Additional ^18^F-fluoroalkylphosphonium derivatives (^18^F-FATPs: ^18^F-FPTP, ^18^F-FHTP, ^18^F-FETP) demonstrated superior image quality and rapid liver/lung clearance compared with ^13^N-ammonia in rat coronary occlusion models (87). These tracers had higher first-pass extraction fractions than ^13^N-ammonia at flow rates exceeding 4.0 mL/min, suggesting improved detection of mild stenosis (88).

#### 3.4.1. ^18^F-Flurpiridaz

Flurpiridaz (formerly [^18^F]BMS-747158 - 02), a structural analog of the pyridazinone insecticide pyridaben (Figure 4), is a mitochondrial complex-1 (MC1) inhibitor that competitively binds the ubiquinone site without affecting cell viability (89). It demonstrates rapid myocardial uptake (time to half-maximal uptake approximately 35 seconds), high retention (9.5% injected dose/g at 60 minutes), slow washout (efflux half-time > 120 minutes), flow-independent first-pass extraction, minimal lung uptake, and rapid hepatic clearance (washout between 5 and 15 minutes) (49, 73, 89-91).

**Figure 4.**
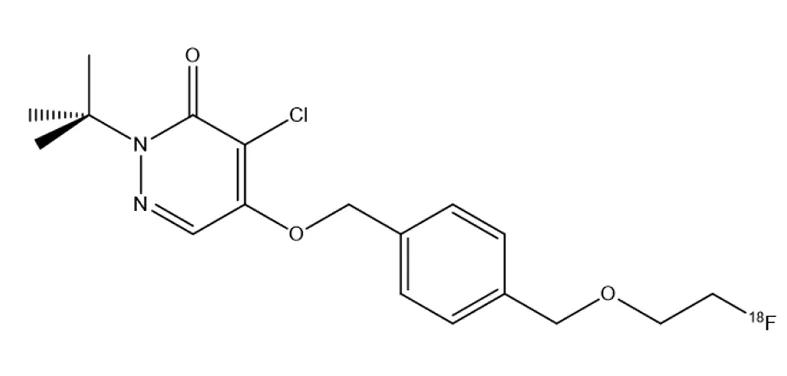
Chemical structure of ^18^F-flurpiridaz

Electrocardiographic safety has been confirmed in both control and myocardial infarction rabbit models (92). Flurpiridaz demonstrates flow-independent first-pass extraction and a linear uptake-flow relationship across a wide flow range (93-99).

#### 3.4.2. Phase I-III Clinical Data

Phase I studies established injected activities of 1.9 mCi (70 MBq) for rest and 6.5 mCi (240 MBq) for exercise stress, or 2.9 mCi (105 MBq) for rest and 6.8 mCi (250 MBq) for pharmacologic stress. Mean effective radiation doses were approximately 4.8 mSv (exercise) and 6.4 mSv (pharmacologic), higher than short-lived tracers ^82^Rb or ^13^N-ammonia (94, 100, 101). The phase II trial demonstrated non-inferiority to ^99^mTc-SPECT and superiority in obese patients. The phase III trial (101, 102) showed significantly higher diagnostic accuracy than SPECT (90.4% vs. 85.6%, P < 0.001), with better sensitivity and specificity. As of 2026, flurpiridaz remains under FDA review and is not yet available for routine clinical use (Table 4) (73, 100-107).

**Table 4.** Advantages and Limitations of ^18^F-Flurpiridaz as a PET Radiotracer for MPI

Advantages	Limitations
**High first-pass extraction of > 90%**	Limited to academic centers
Almost linear correlation between tracer uptake and myocardial blood flow in isolated perfused rat heart	Cyclotron production
**Radiotracer redistribution after reperfusion**	No FDA approval yet
Phase I: radiotracer present up to 5 h after injection, i.e., injection at peak treadmill exercise is feasible	Cost-effectiveness data are lacking
Phase II: compared to stress-rest SPECT MPI, PET MPI with superior performance characteristics for overall CAD diagnosis	-
First Phase III study: superior perfusion defect detection of Flurpiridaz relative to SPECT MPI for the assessment of CAD in obese subjects; efficacy and high sensitivity of Flurpiridaz PET MPI in detecting significant CAD compared to SPECT MPI in patients scheduled for invasive coronary angiography	-

### 3.5. Comprehensive Comparison of SPECT and PET MPI Tracers

The following table provides a comprehensive comparison of all SPECT and PET MPI tracers using consistent evaluation criteria (Table 5) (93):

**Table 5.** Comprehensive Comparison of SPECT and PET Myocardial Perfusion Tracers

Characteristic	^201^Tl	^99^mTc-Sestamibi	^99^mTc-Tetrofosmin	^82^Rb	^13^N-Ammonia	^15^O-Water	^18^F-Flurpiridaz
**Half-life**	73 h	6 h	6 h	76 s	10 min	2 min	110 min
**Positron Range (mm)**	N/A	N/A	N/A	8.60	2.53	4.14	1.03
**Myocardial Extraction (%)**	85	65	60	65	80	100	94
**Production**	Cyclotron	Generator	Generator	Generator	Cyclotron	Cyclotron	Cyclotron
**Exercise Stress**	Yes	Yes	Yes	No	No	No	Yes
**Absolute MBF Quantification**	No	No	No	Yes	Yes	Yes	Yes
**Effective Dose (mSv)**	34.5	6.3	5.0	2.0 - 3.7	1.3 - 2.0	1.0 - 1.5	4.8 - 6.4
**Spatial Resolution**	Poor	Moderate	Moderate	Moderate	Good	Good	Excellent
**Attenuation Correction**	Limited	Limited	Limited	Routine	Routine	Routine	Routine
**Availability**	Widely	Widely	Widely	Moderate	Limited	Research	Investigational
**Regulatory Status**	FDA/EU	FDA/EU	FDA/EU	FDA/EU	FDA/EU	Research	Phase III
**Cost**	Low	Moderate	Moderate	High	High	Very High	Moderate

## 4. Conclusions

The updated evidence supports a complementary model of PET and SPECT MPI. ^99^mTc-sestamibi and ^99^mTc-tetrofosmin remain clinically established SPECT agents with major advantages in availability, workflow, and cost. CZT detector technology and modern attenuation correction have improved the performance of contemporary SPECT and can reduce acquisition time or administered activity. PET offers higher sensitivity, routine attenuation correction, and quantitative MBF/MFR; however, these benefits depend on appropriate dynamic acquisition, kinetic modeling, motion correction, and tracer availability.

The major recent radiopharmaceutical development is the clinical translation of ^18^F-flurpiridaz. Its 2024 U.S. approval changes the previous regulatory landscape and establishes a longer-lived ^18^F perfusion tracer as a clinical option for rest and stress PET MPI, including exercise stress. Its clinical value should nevertheless be considered in the context of local regulatory status, reimbursement, production and distribution capabilities, and the availability of validated quantitative analysis.

Several technical issues remain important. Positron range contributes to the lower intrinsic spatial resolution of high-energy emitters such as ^82^Rb compared with ^18^F. PET quantitative accuracy can be affected by partial-volume and blood-pool spillover, dynamic framing, attenuation misregistration, and motion. SPECT remains vulnerable to photon attenuation and extracardiac activity, although CT attenuation correction, prone imaging, CZT detectors, iterative reconstruction, and emerging machine-learning approaches can mitigate these limitations.

### 4.1. Limitations

This review has limitations. It is a narrative rather than a systematic review; no formal risk-of-bias assessment or meta-analysis was performed, and study selection and synthesis were author-directed. Regulatory availability and reimbursement are time- and jurisdiction-dependent and should be verified locally before clinical implementation. Future comparative research should emphasize standardized MBF/MFR protocols, multicenter diagnostic validation, health-economic analysis, patient-specific acquisition, and integration of quantitative PET with anatomic and clinical risk information. The evidence therefore supports a role for both modalities rather than a universal replacement of SPECT by PET.

## Data Availability

The dataset presented in the study is available on request from the corresponding author during submission or after publication.
